# Unidirectional Polyvinylidene/Copper-Impregnated Nanohydroxyapatite Composite Membrane Prepared by Electrospinning with Piezoelectricity and Biocompatibility for Potential Ligament Repair

**DOI:** 10.3390/polym17020185

**Published:** 2025-01-14

**Authors:** Chih-Hsin Cheng, Wen-Cheng Chen, Wen-Chieh Yang, Sen-Chi Yang, Shih-Ming Liu, Ya-Shun Chen, Jian-Chih Chen

**Affiliations:** 1 Clinical Histopathology Division, Hualien Armed Forces General Hospital, Hualien 970, Taiwan; allen158170@yahoo.com.tw; 2 Advanced Medical Devices and Composites Laboratory, Department of Fiber and Composite Materials, Feng Chia University, Taichung 407, Taiwan; a23581541peter@gmail.com (W.-C.Y.); bob410487@gmail.com (S.-C.Y.); 0203home@gamil.com (S.-M.L.); ya0610ya@gmail.com (Y.-S.C.); 3 Department of Fragrance and Cosmetic Science, College of Pharmacy, Kaohsiung Medical University, Kaohsiung 807, Taiwan; 4 Dental Medical Devices and Materials Research Center, College of Dental Medicine, Kaohsiung Medical University, Kaohsiung 807, Taiwan; 5 Department of Orthopedics, Faculty of Medical School, College of Medicine, Kaohsiung Medical University, Kaohsiung 807, Taiwan; 6 Department of Orthopaedics, Kaohsiung Medical University Hospital, Kaohsiung 807, Taiwan

**Keywords:** electrospinning, nanoparticles, composite membranes, piezoelectricity

## Abstract

Ligament tears can strongly influence an individual’s daily life and ability to engage in physical activities. It is essential to develop artificial scaffolds for ligament repairs in order to effectively restore damaged ligaments. In this experiment, the objective was to evaluate fibrous membranes as scaffolds for ligament repair. These membranes were created through electrospinning using piezoelectric polyvinylidene fluoride (PVDF) composites, which contained 1 wt.% and 3 wt.% of copper-impregnated nanohydroxyapatite (Cu-nHA). The proposed electrospun membrane would feature an aligned fiber structure achieved through high-speed roller stretching, which mimics the properties of biomimetic ligaments. Nanoparticles of Cu-nHA had been composited into PVDF to enhance the pirzoelectric β-phase of the PVDF crystallines. The study assessed the physicochemical properties, antibacterial activity, and biocompatibility of the membranes in vitro. A microstructure analysis revealed that the composite membrane exhibited a bionic structure with aligned fibers resembling human ligaments. The piezoelectric performance of the experimental group containing 3 wt.% Cu-nHA was significantly improved to 25.02 ± 0.68 V/g·m^−2^ compared with that of the pure PVDF group at 18.98 ± 1.18 V/g·m^−2^. Further enhancement in piezoelectric performance by 31.8% was achieved by manipulating the semicrystalline structures. Antibacterial and cytotoxicity tests showed that the composite membrane inherited the antibacterial properties of Cu-nHA nanoparticles without causing cytotoxic reactions. Tensile tests revealed that the membrane’s flexibility of strain was adequate for use as artificial scaffolds for ligaments. In particular, the mechanical properties of the two experimental groups containing Cu-nHA were significantly enhanced compared with those of the pure PVDF group. The favorable piezoelectric and flexible properties are highly beneficial for ligament tissue regeneration. This study successfully developed PVDF/Cu-nHA piezoelectric fibers for a biocompatible, unidirectional piezoelectric membrane with potential applications as ligament repair scaffolds.

## 1. Introduction

When the anterior cruciate ligament (ACL) is damaged or ruptured, the knee joint loses stability, rendering the injured person unable to stand and walk normally. ACL damage can occur because the ACL is located at the junction of bones and has no blood vessels. Its self-healing capability is limited, making artificial scaffolds necessary for ACL repair [1,2,3]. At present, most artificial scaffolds for ligament repairs are made of polymers with biocompatibility and good mechanical strength. However, attention should be paid to the aging fatigue of the materials, which can cause joint hydration and inflammation. Furthermore, artificial scaffolds are affixed and transplanted into the bone tunnels drilled in the tibia. As such, these artificial scaffolds must possess adequate mechanical strength and promote osseointegration between the ligament and the bone tunnel [4].

Electrospun membranes composed of ultrafine fibers have the advantages of a high specific surface area, high porosity, and simulated extracellular matrix (ECM), providing cells with a good attachment and growth-guiding environment. Unidirectional nanofibers produced by electrospinning have similar properties to the natural ligaments of the collagen fiber matrix [5,6,7]. Furthermore, bundling the fibers, extending the electrospinning process duration, or laminating aligned sheets can result in greater yield stress compared to using a single sheet. The mechanical deformation strain generated by unidirectional fibers can help the fibroblasts in the ligament grow directionally, increase ECM production, and bring the new tissue closer to the natural ligament structure. Orthotropic fibers also have strong directional mechanical properties and can match the mechanical properties of human ligaments.

Some biological tissues, such as bones, ligaments, and corneas, have natural piezoelectric properties. The surface polarization and electrical signals from the piezoelectric effect can attract cells and regulate ions in organisms. This phenomenon affects intracellular ion concentration, influences gene expression, and accelerates cell differentiation, proliferation, and migration, thereby promoting the regeneration of target tissues [8,9]. Polyvinylidene fluoride (PVDF) is a piezoelectric polymer known for its high piezoelectric coefficient. This property enables it to mimic natural piezoelectric tissues, promote the expression of regeneration factors, facilitate cell attachment to PVDF, and enhance cell differentiation and proliferation. As a result, PVDF is an excellent choice for artificial scaffolds for ligament regeneration, providing effective repair outcomes [10,11].

PVDF is a thermoplastic semicrystalline polymer synthesized from 1,1-vinylidene fluoride via free radical polymerization. It exhibits excellent piezoelectricity, flexibility, and biocompatibility, which enhance cell proliferation and differentiation, thereby promoting tissue regenerations. PVDF has a semicrystalline nature due to the flexibility of its chains and has five different crystalline forms: α, β, γ, δ, and ε. The α and ε forms are nonpolar phases, and the β, γ, and δ forms exhibit ferroelectric polarity [12]. In particular, the β-phase among stereoisomers has an all-trans conformation, i.e., fluorine atoms and hydrogen atoms are separately arranged on both sides of the molecular chain, creating a polar moment coupled with excellent piezoelectricity. The stretching and elongation caused by an electrified polymer jetting during electrospinning can enhance the crystalline component of the β-phase of PVDF, thereby improving the piezoelectricity [13,14,15]. Although PVDF has good biocompatibility and can promote cell growth, it cannot induce osteogenesis. Therefore, adding some factors that help PVDF induce osteogenesis is necessary to enhance the osseointegration effect and securely fix the artificial scaffolds with ligaments into the bone tunnel.

Nanohydroxyapatite (nHA) has excellent osteoconductive properties, promoting the regeneration of bone tissues and facilitating the osseointegration of artificial ligaments. Impregnating copper ions into nHA (Cu-nHA) nanoparticles makes them antibacterial, reduces subsequent complications, and enhances the effect of ligament repair. The Cu^2+^ in nHA has a synergistic effect that can accelerate the release of Ca^2+^ and increase the concentration of free Ca^2+^ in the solution, which is beneficial to osseointegration. Additionally, adding metal-impregnated nanoparticles doped with metal ions can improve the piezoelectricity of PVDF [16,17,18,19,20].

In this experiment, PVDF served as the polymer matrix for the piezoelectric membrane, while Cu-nHA nanoparticles acted as the additive. An alternative hypothesis was proposed to investigate the piezoelectric and antibacterial properties of the composite membrane. This experiment aimed to develop fibrous membranes and evaluate the efficacy as scaffolds for ligament repair. These membranes were developed through electrospinning piezoelectric polyvinylidene fluoride (PVDF) fibers, which incorporated 1 wt.% and 3 wt.% of Cu-nHA nanoparticles. The designed electrospun membrane featured an aligned fiber structure achieved through high-speed roller stretching, which emulates the characteristics of biomimetic ligaments. The integration of Cu-nHA nanoparticles into the PVDF aimed to enhance the piezoelectric β-phase of the crystalline structure of PVDF. This study examined the membranes’ physicochemical properties, antibacterial activity, and biocompatibility in vitro. The microstructural analysis demonstrated that the composite membrane exhibited a biomimetic structure with aligned fibers resembling human ligaments. The enhancement in piezoelectricity may facilitate ligament tissue regeneration and improve the integration of the artificial ligament with the bone interface.

## 2. Materials and Methods

### 2.1. Raw Materials

The raw materials used for synthesizing Cu-nHA nanoparticles were calcium nitrate (Ca(NO_3_)_2_∙_4_H_2_O, purity > 98.0%, KATAYAMA CHEMICAL Co., Ltd., Osaka, Japan), diammonium phosphate ((NH_4_)_2_HPO_4_, purity > 98.0%, HSE PURE CHEMICALS, Ahmedabad, India), sodium citrate (Na_3_C_6_H_5_O_7_, purity > 99.0%, PANREAC, Barcelona, Spain), and copper (II) nitrate (Cu(NO_3_)_2_∙_3_H_2_O, purity > 99.0%, PANREAC, Barcelona, Spain). Other materials used included acetone (99.5%, Panreac, Barcelona, Spain), N,N-dimethylformamide (DMF, >99.0%, Sigma–Aldrich, St. Louis, MO, USA), and PVDF in powder form, also sourced from Sigma–Aldrich, as a fiber matrix with a molecular weight of approximately 534,000 g/mol.

### 2.2. Preparations of Cu-nHA and PVDF/Cu-nHA Nanofibers

#### 2.2.1. Cu-nHA Nanoparticles

To synthesize Cu-nHA nanoparticles, the hydrothermal method was applied. First, 0.11 g copper nitrate and 2.11 g calcium nitrate were dissolved into 90 mL of deionized water, and the pH was adjusted to 7 to obtain the first solution. Afterward, 2.65 g of sodium citrate and 0.71 g of diammonium hydrogen phosphate were added to 70 mL of deionized water to obtain a second solution. The two solutions were mixed and stirred for 30 min before being heated to 180 °C through a hydrothermal method for 12 h (SP88857100, Thermo Fisher Scientific, Waltham, MA, USA). The precipitate was washed three times with alcohol and deionized water, then filtered and dried in an oven at 60 °C to obtain Cu-nHA particles. The morphologies of the Cu-nHA nanoparticles were observed using transmission electron microscopy (TEM; JEM-2100F, JEOL, Tokyo, Japan). Subsequently, Image J2 software (Wisconsin, Madison, WI, USA) was utilized to analyze the length, width, and aspect ratio of the Cu-nHA nanoparticles.

#### 2.2.2. Electrospun PVDF/Cu-nHA Nanofiber Preparation

A 1:1 ratio of DMF and acetone was prepared as the solvent, to which 0, 1, and 3 wt.% of Cu-nHA particle additives were subsequently added. The Cu-nHA particles were uniformly dispersed in the solvent using a 40 kHz ultrasonic vibration (Delta DC400H, Taoyuan, Taiwan) to create a stable suspension without aggregation. Finally, 15 wt.% of PVDF powder was incorporated into the Cu-nHA solution by heating the mixture to 60 °C for 1 h under constant magnetic stirring. This process resulted in a PVDF/Cu-nHA electrospinning solution.

The PVDF electrospinning solutions were prepared without and with 1 wt.% and 3 wt.% Cu-nHA nanoparticle concentrations. These solutions were then poured into a 10 mL syringe, which was attached to a micro-pusher (YSP-101, YMC Taiwan Co., Ltd., Taipei, Taiwan) and set to a push speed of 1.2 mL/h. The high-voltage power supply (AU-60P1.6, Matsusada Precision, Shiga, Japan) was set to 13 kV with a working distance of 10 cm. The electrospun fibers were collected on a drum collector with a diameter of 16 cm, operating at a linear speed of 17 m/s. The electrospun fibrous membranes of PVDF/Cu-nHA were obtained after 6 h of collection.

### 2.3. Characterized Physiochemical Properties

#### 2.3.1. Micromorphology and Fiber Diameter Measurement

A 1 cm × 1 cm electrospun membrane was adhered to a metal plate with carbon tape and coated with conducting metal. The fiber morphology and structure of the membranes were observed under a scanning electron microscope (SEM, S-3400N, Hitachi, Tokyo, Japan). Elemental mapping was used to detect Cu-nHA distribution on the fibers. Finally, Image J was used to calculate the average fiber diameter in each group.

#### 2.3.2. F(β) Index Calculation Through Infrared Spectra

Nanofiber membranes are naturally opaque, so an attenuated total reflectance Fourier transform infrared spectrometer (ATR-FTIR, Nicolet iS5, Thermo Fisher Scientific, Waltham, MA, USA) was used to detect the resulting piezoelectric β-phase of PVDF in fibers. The relative content of the β-phase can be analyzed from the ATR-FTIR analysis as one of the important indicators for judging piezoelectric performance. The calculation formula was derived from the Beer–Lambert law of light absorption as follows [21,22]:

$$F\left(\beta \right)=\frac{A_{\beta }}{\left(\frac{K_{\beta }}{K_{\alpha }}\right)A_{\alpha }+A_{\beta }}$$

where (K_β_/K_α_) is a constant value of 1.3. A_α_ represents the α absorption at a wavelength of 761 cm^−1^, and A_β_ denotes the β absorbance at 838 cm^−1^.

#### 2.3.3. Piezoelectric Response Under Dynamic Bending

**Figure 1:**
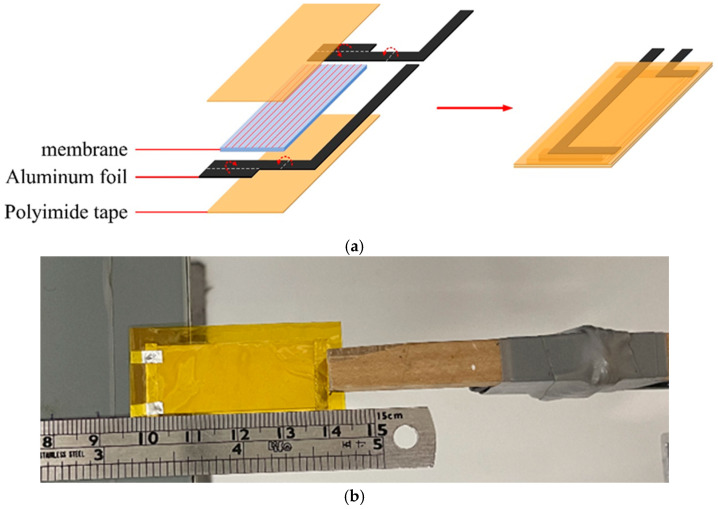
A schematic of a piezoelectric test specimen of the packaging test piece preparation (**a**) and image of the packaging test piece under the dynamic bending setup for piezoelectic responses (**b**).

The membrane was cut into a rectangle measuring 4.0 cm by 1.5 cm. Two pieces of aluminum foil were used as conductors, and a 1.0 cm by 0.3 cm rectangle served as the testing electrode. The aluminum foil was positioned vertically on either side of the membrane, and the electrode was placed symmetrically on one side. Finally, polyimide tape was applied to the outer surface of the sample to protect it from external environmental charges and create a piezoelectric test piece (Figure 1a). For the piezoelectric test, the sample was fixed flatly on a horizontal base. A contact was made between the oscilloscope probe and the aluminum foil electrode prepared in advance, and the rotation frequency of the tapping device was set to 1 Hz. The bending point of the specimen between the tapping points was fixed at 1.6 cm, and tapping was conducted eight times (Figure 1b). The voltage (peak-to-peak) generated on the oscilloscope during the repeated deformation of the specimen was measured and recorded for subsequent calculation.

#### 2.3.4. Tensile Measurement

In accordance with the ASTM D882 specification [23], the membrane was cut into dumbbell-shaped specimens and stretched along the longitudinal direction of the fibers. The thickness of each specimen was measured and a tensile test was performed at a rate of 5 mm/min using a universal testing machine (HT-2402, Hung Ta, Taichung, Taiwan) equipped with a 200 N load cell. The tensile stress versus strain curve changes were recorded until the sample ruptured.

### 2.4. Antibacterial Tests

The study focused on quantitative antibacterial testing using two bacterial strains: Gram-positive *Staphylococcus aureus* (*S. aureus*; ATCC number: 25923) and Gram-negative *Escherichia coli* (*E. coli*; ATCC number: 10798) [24]. The culture environment for these strains was maintained at 37 °C without CO_2_ to prevent bacterial overgrowth. Prior to the experiments, the electrospun aligned fibrous membranes of pure PVDF, PVDF/1 wt.% Cu-NHA, and PVDF/3 wt.% Cu-nHA were subjected to UV sterilizations.

Antimicrobial quantification was conducted using a broth dilution method. First, the bacterial suspension was diluted to an optical density (OD) of 0.2 at OD_595_, resulting in an average concentration of approximately 1.0 × 10^7^ cells/mL. Next, the sterilized sample was immersed in 2 mL of the bacterial suspension and incubated at 37 °C for 1 day. After incubation, 100 µL of the bacterial suspension was transferred to a 96-well plate. The absorbance value at OD_595_ was then measured using an enzyme-linked immunosorbent assay (ELISA) reader (EZ Read-400, Biochrom, Holliston, MA, USA) to evaluate the antibacterial effect.

### 2.5. In Vitro Cytotoxicity Tests

All the cells were cultured in an incubator with 5% CO_2_ at 37 °C. The neonatal mouse fibroblast L929 cell line (National Institute of Health, Miaoli, Taiwan) was used for the cytotoxicity assay. Minimal Essential Medium alpha (Gibco, Thermo Fisher Scientific Inc., Waltham, MA, USA) containing 10% horse serum was employed as a culture medium and changed every 2 days of culture. The L929 cells were subcultured at a cell concentration of 0.8 × 10^6^ cells/mL to 1.0 × 10^6^ cells/mL. All the samples used for the subsequent in vitro cell culture were sterilized under γ-radiation at 25 kGy (China Biotechnology Co., Ltd., Taichung, Taiwan). The test procedures followed the ISO 10993-5:2009 guidelines [25]. Pure PVDF and PVDF with 1 wt.% and 3 wt.% Cu-nHA membranes were soaked in a cell culture medium at a ratio of 6 cm^2^ to 1 mL and placed in a 37 °C incubator for 24 h. The culture medium was then extracted for the cytotoxicity test. The control group consisted of a blank cell culture medium. The positive control group was treated with 15 vol.% dimethyl sulfoxide (Sigma-Aldrich, St. Louis, MO, USA), and the negative control group was treated with high-density polyethylene that was subjected to the same sterilization process as the experimental group. Hence, the sterilization status of the experimental group could be observed.

In the quantitative cytotoxicity test, each group of extracts (100 μL per well) was added to 1 × 10^4^ L929 cells. The cells were then cultured for 24 h. The medium was removed, and 100 μL of a fresh medium was mixed with 50 μL of the XTT cell proliferation assay kit (Biological Industries, Kibbutz Beit Haemek, Israel) for a 4 h reaction. The samples were analyzed using an ELISA reader (SPECTROstar Nano, BMG LABTECH, Offenburg, Germany). The absorbance measured from OD492 is directly proportional to the cell viability.

The same extract was used for the qualitative cytotoxicity test to treat L929 cells (1000 μL per well) with a cell concentration of 1 × 10^5^ cells in each extract group. The cells were cultured for 24 h, and their morphology was observed.

### 2.6. Statistical Analysis

IBM SPSS Statistics 20 (SPSS Inc., Chicago, IL, USA) was used to analyze the data. An F(β) index analysis and piezoelectric dynamic bending testing were conducted to evaluate significant differences. Tukey’s test was also used for pairwise post hoc testing to determine any differences between the mean of all pairs, with a *p*-value of less than 0.05 indicating statistical significance.

## 3. Results and Discussion

### 3.1. Microstructures and Average Fiber Diameter of Electrospun Membranes

**Figure 2:**
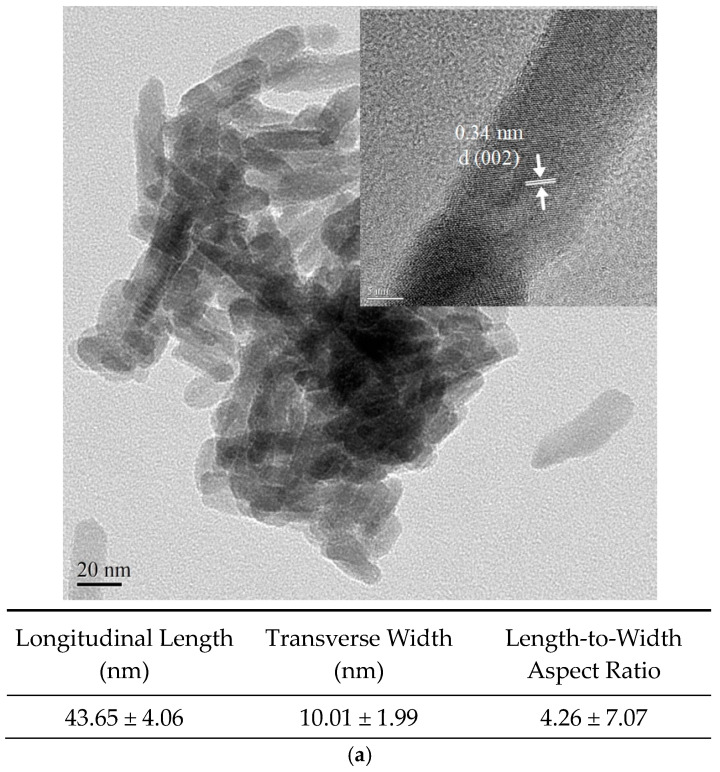

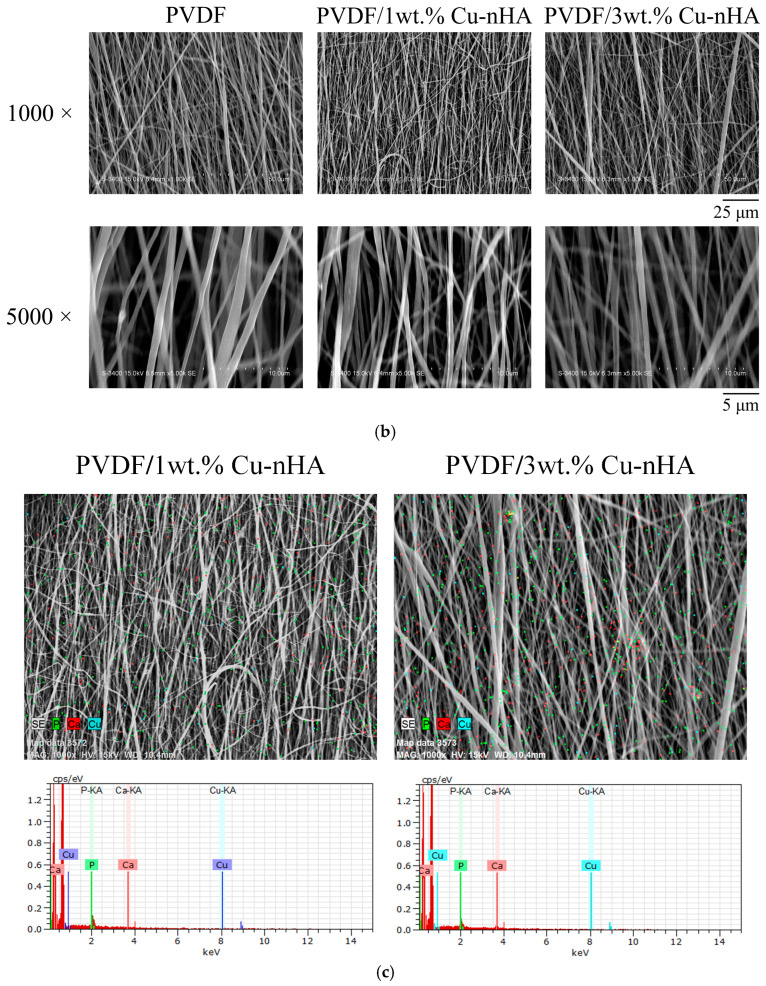
(**a**) The analysis of TEM images of Cu-nHA nanoparticles, focusing on their length, width, and aspect ratio (*n* = 20); (**b**) microstructural images of each electrospun fibrous membrane; (**c**) analyzing the element mapping to ensure the distribution of Cu-nHA nanoparticles within electrospun PVDF fibers (green: P, red: Ca, and blue: Cu).

The characteristics of the Cu-nHA rod-shaped nanoparticles are shown in Figure 2a. The observed lattice distance of 0.34 nm corresponds to the HA(002) diffraction plane. The average dimensions of the Cu-nHA nanoparticles are 43.65 nm in length and 10.01 nm in width, resulting in an aspect ratio of 4.26. Using the electrospinning method described above, fibrous membranes were successfully fabricated reproducibly. Microstructural observations reveal that the surface of each fiber group is smooth and uniform, with no polymer beads present (see Figure 2b). This indicates that Cu-nHA nanoparticles are well dispersed within the PVDF nanofibers. The nanofibers are aligned and oriented along the fiber axis, showing no tangling. Table 1 presents the average fiber diameter results for the aligned electrospun PVDF without and with Cu-nHA nanoparticles, and includes a further quantitative analysis of 100 fibers from the image. With the increase in Cu-nHA concentration, the fiber average diameters of the PVDF incorporated with 1 wt.% and 3 wt.% Cu-nHA show a significant downward trend (*p* < 0.05). This phenomenon can be attributed to the addition of Cu-nHA nanoparticles, which enhances the conductivity of the electrospinning solution and subjects it to a stretching force from the electric field during the electrospinning [26]. Literature comparison revealed that the average fiber diameter of PVDF/3 wt.% is comparable to that of collagen fibers found in dense connective tissues. This finding indicated that the fiber morphology of PVDF/3 wt.% Cu-nHA resembles that of human ligaments [27].

The topographical structure plays an important role in signaling stem cell differentiation. Many studies have documented how microtopography and nanotopography affect cell behavior. In particular, parallel-aligned topography is crucial for promoting tenogenic, neurogenic, and myogenic differentiation [12,28,29].

Zhou et al. [28] established an in vitro tendon engineering model, in which mouse adipose stem cells were seeded onto aligned miniaturized polyglycolic acid fibers. After 8 weeks of culture, these cells can partially regenerate new tendon tissues, forming well-arranged collagen fibers in the surrounding area. Polarized light microscopy confirmed the relative maturity of the collagen fibers. The results showed that micro- and nanoscale topologies with parallel alignment can effectively drive the tendon differentiation of seeded human adipose stem cells.

Figure 2c shows the elemental mapping analysis of both PVDF/1 wt.% and 3 wt.% Cu-nHA. Calcium, phosphorus, and copper are present and evenly distributed in both sets of membranes. This finding demonstrated the effective integration of Cu-nHA nanoparticles in PVDF, resulting in a uniform distribution of the nanoparticles.

### 3.2. IR Spectral and F(β) Index Analysis

**Figure 3:**
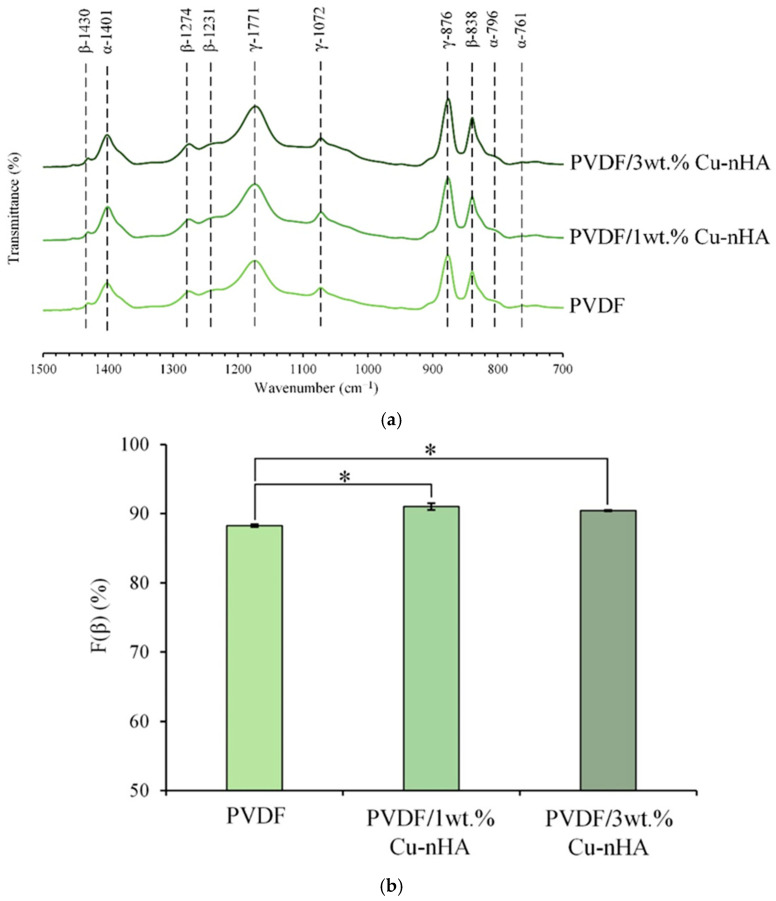
(**a**) ATR-FTIR spectra of each group of electrospun membranes; (**b**) the corresponding F(β) index analysis (*n* = 3, * indicates a *p*-value of less than 0.05 with a significant difference between the groups).

The IR analysis of each group is shown in Figure 3a. The characteristic bands of the α-phase of PVDF crystallization are at wavelengths of 761, 796, and 1401 cm^−1^, and the absorptions of the β-phase are at wavelengths of 838, 1231, 1274, and 1430 cm^−1^. The characteristics of the γ-phase are at wavelengths of 876, 1072, and 1171 cm^−1^. Figure 3b illustrates each electrospun membrane’s F(β) index analysis, focusing on the characteristic peaks of the α- and β-phases obtained from the spectra. The F(β) indexes for the three groups of pure PVDF and PVDF with 1 wt.% and 3 wt.% Cu-nHA are 88.27%, 91.02%, and 90.43%, respectively. This result indicated that the gradual addition of Cu-nHA nanoparticles enhances the β-phase of PVDF, leading to an increased presence of piezoelectric phases. The increase in Cu-nHA nanoparticles may enhance the effective stretching of the PVDF fiber during the electrospinning under an applied electric field. This phenomenon reduces fiber diameter and increases the stretch-enhanced polarization of the β-phase in the PVDF fibers [30,31].

### 3.3. Piezoelectric Voltage Generated by Electrospun Membranes Under Dynamic Bending

The piezoelectric properties of each membrane are presented in Table 2. Notably, the piezoelectric value significantly increases when the amount of Cu-nHA nanoparticles rises to 3 wt.%. In contrast, a decrease in the piezoelectric value is observed in the group with 1 wt.% Cu-nHA. This phenomenon indicates that introducing a small quantity of nanoparticles can adversely affect the internal stereochemical configuration of PVDF-based fibers. This negative impact results from the uneven interactions between the conductive Cu-nHA nanoparticles and the PVDF molecules. If there are too few nanoparticles, they may either remain trapped within the fiber or accumulate on its surface, leading to the disruption of the PVDF’s stereochemical configuration [32]. This damage reduces the charge released during fiber deformation, resulting in a decrease in the piezoelectric value. When enough nanoparticles are added, the negative effects of structural damage are counterbalanced by the additional piezoelectric effect generated by Cu-nHA, which promotes polarization [26,33,34]. As a consequence, the overall piezoelectricity of the material is enhanced.

Direct and reverse piezoelectric energy conversions are applied in various biological fields, such as biosensors, bioactuators, and drug delivery systems [35]. The natural bioelectrical activity and piezoelectric properties found in various body tissues—such as nerves, bones, dentin, cartilage, and ligaments—have prompted great efforts to utilize piezoelectric materials as self-powered platforms for electrically stimulating cells and tissues. This approach is gaining traction in regenerative medicine and tissue engineering.

Tai et al. [36] demonstrated that electrospinning improves the piezoelectric properties of P(VDF-TrFE) nanofibers, and this enhancement is influenced by the fiber size. The improvement occurs due to the changes in the content of the electroactive phase and the alignment of piezoelectric domains, which are affected by the high electric fields and mechanical stretching during electrospinning. They revealed that electrospinning allows PVDF to align its dipoles perpendicular to the fiber direction, promoting a transition from the amorphous phase to crystalline phases.

In the F(β) index test, there is no significant difference between the two groups with different concentrations of Cu-nHA added to PVDF; however, both groups perform better than the pure PVDF group. Notably, the addition of 3 wt.% Cu-nHA to PVDF yields better piezoelectric values compared to the addition of 1 wt.% Cu-nHA. The results from both tests indicate that incorporating nanoparticles increases the β-phase of PVDF, and that a higher concentration of these nanoparticles effectively enhances the piezoelectric properties of the membrane. The presence of Cu-nHA nanoparticles may promote a higher β-phase orientation in PVDF, resulting in an increased voltage output.

### 3.4. Tensile Testing of Membranes

**Figure 4:**
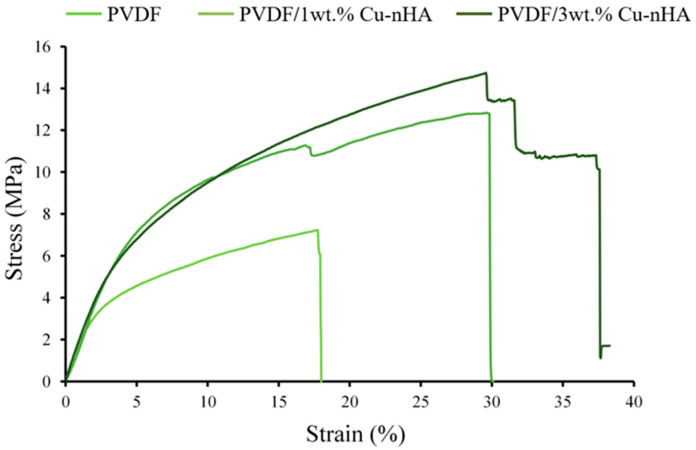
Typical stress–strain curves of electrospun membranes showing tensile load displacement in longitudinal tensile tests.

The representative tensile testing results presented in typical stress–strain curves of aligned electrospun PVDF and PVDF with 1 and 3wt.% Cu-nHA nanofibrous membranes are illustrated in Figure 4. At an initial strain load of less than 2%, the curves of the three membranes show approximately the same modulus because the matrix of the nanofiber mesh is the same as that of PVDF when tensile force is applied. The addition of Cu-nHA significantly enhanced the mechanical properties of the membranes, such as elastic yield stress, toughness, and fracture staining, especially for the highest hybrid dose of 3 wt.% Cu-nHA nanoparticles. Table 3 shows the mechanical properties of each electrospun membrane. The measured maximum tensile stress and strain of the PVDF/1 wt.% and PVDF/3 wt.%, the breakage.% of membranes with Cu-nHA nanoparticles were approximately twice that of the pure PVDF membrane. This finding confirms that the addition of Cu-nHA nanoparticles can significantly enhance the mechanical properties of aligned electrospun membranes. Notably, the elongation at break of PVDF/Cu-nHA nanofibrous membranes did not decrease, and even the stiffness of the aligned fiberous membrane did not decrease with the addition of nanoparticles as in the case of randomly oriented PVDF [21,23]. The flexibility of electrospun piezoelectric nanofiber polymer-based membranes is similar to that of biological tissues due to their stretchable properties. The ACL is frequently ruptured by traumatic loading, and a large number of ACL tears occur each year and the ACL posterolateral bundle is 15% and tensile strength is 15 MPa [37]. Additionally, the structure of the aligned electrospun nanofibers mimics the dimensions of the collagen fibrils that make up natural tendons and ligaments [4]. Notably, the results of this study suggest that aligned nanofibrous membranes may be suitable for use as tissue-engineering scaffolds because the modulus and yield properties of the scaffolds meet the mechanical requirements of the native ACL of approximately 27~30% [38]. Altering the orientation and structure of nanofibers can significantly impact their mechanical properties. For instance, aligned nanofiber sheets exhibit a higher modulus than randomly arranged nanofiber sheets. Additionally, creating thicker nanofiber membranes by laminating aligned sheets can achieve more significant yield stresses; however, the strain observed will not exceed that of a single sheet. This resemblance helps prevent tissue damage from mechanical mismatches between implanted piezoelectric materials and the surrounding biological tissues. In addition, piezoelectric nanofibers can mimic the biological functions of collagen fibers found in the ECM. These collagen fibers serve as a natural material for electromechanical conversion and facilitate the transmission of bioelectric signals, enabling communication between cells [39,40,41]. Thus, the tensile strain after rupture of the PVDF/Cu-nHA membrane groups meets the deformation requirements for the ACL, indicating their potential for repair or replacement.

### 3.5. Antibacterial Testing

**Figure 5:**
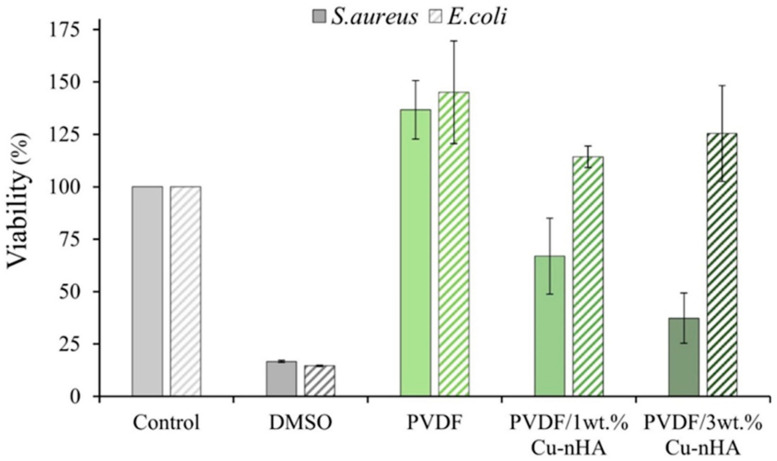
The quantitative test on the antibacterial activity of each electrospun membrane on *S. aureus* and *E. coli* after 1 day of culture (*n* = 3).

The results of quantitative antibacterial tests for each electrospun membrane against *E. coli* and *S. aureus* after 24 h of culture are presented in Figure 5. The control group, consisting of PVDF, shows no antibacterial effect against either *E. coli* or *S. aureus*. The experimental groups, which include PVDF/1 wt.% and /3 wt.% Cu-nHA, also exhibit no antibacterial activity against *E. coli*. However, these two groups show antibacterial effects of 33.02% ± 18.14% and 62.65% ± 11.92%, respectively, against *S. aureus*. The antibacterial effectiveness of copper ions primarily relies on a contact-based mechanism. When copper ions (Cu^2^⁺) come into contact with bacteria, they can break down the bacteria’s cell walls and penetrate their interior. This phenomenon disrupts the bacteria’s biochemical processes and leads to the generation of highly reactive oxygen species (ROS), ultimately damaging the internal structure of the bacteria and leading to their death. However, *E. coli* possesses a complex and resistant cell wall structure, making it challenging for copper ions to penetrate and activate their antibacterial properties within *E. coli* cells [42,43]. The cell wall structure of *S. aureus* is relatively simple, facilitating the easy penetration of Cu^2^⁺ ions. This penetration leads to an effective antibacterial mechanism. The Cu^2^⁺ released from membrane extracts can activate antibacterial properties against *E. coli*, presenting advantages over membranes that do not release Cu^2^⁺. In addition, the antibacterial properties of Cu^2^⁺ help reduce the complications associated with bacterial infections, particularly during the early stages of transplanting artificial grafts into the human body. Aside from the type of bacteria, the bactericidal effect of copper-containing hydroxyapatite powder also depends on its quantity. Nouri et al. [24] observed that bacteria can thrive in the presence of 50 mg/mL copper-containing hydroxyapatite. As a consequence, the success rate of surgeries is significantly improved, and the compatibility between artificial grafts and the human body is enhanced. Further studies on the enhancement of Cu^2^⁺ release may reveal a synergistic effect involving Cu^2^⁺ and piezoelectricity in vitro and in vivo, which should be explored in future research.

### 3.6. Cytotoxicity Testing

**Figure 6:**
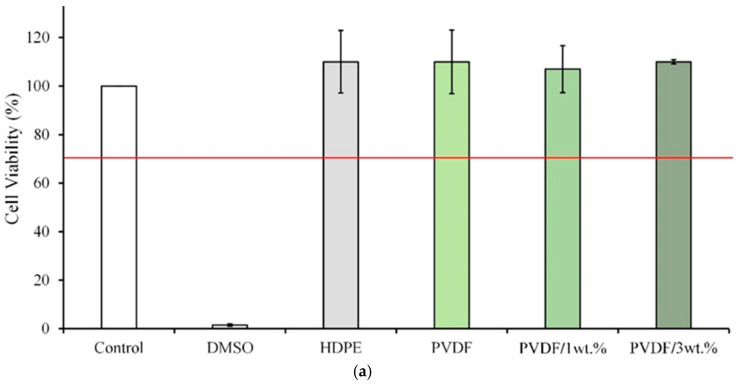

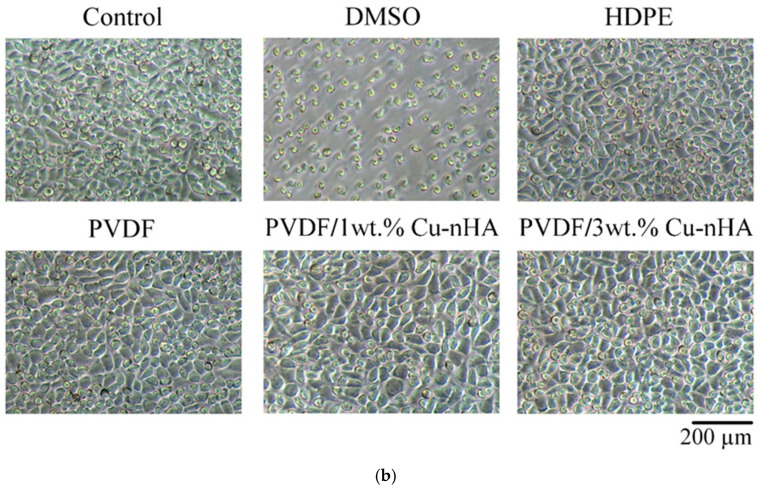
Electrospun membrane extracts of each group cultured with L929 cells for 1 day: (**a**) quantitative and (**b**) qualitative cytotoxicity test (*n* = 6); The red line in the cell viability test indicates that viability exceeds 70%, suggesting the substance may not be cytotoxic compared to the control group.

According to ISO10993-5 specifications, a material extract is toxic to cells when the cell viability is lower than 70% or the cell morphology changes significantly. Figure 6a presents the quantitative analysis of each extract and L929 cells cultured for 1 day. The cell survival rate in the PVDF/1 wt.% and PVDF/3 wt.% Cu-nHA groups exceeds 70%, suggesting no significant cytotoxicity. Figure 6b shows the cell morphology of L929 cells cultured with PVDF/1 wt.% and PVDF/3 wt.% Cu-nHA extracts for 1 day. Both groups have similar effects to the control group, indicating that the cells grow well and are not cytotoxic. These results indicate that incorporating Cu-nHA into electrospun PVDF membranes can reduce the risk of complications and immune rejection following transplant surgery. The piezoelectric effect refers to the conversion of mechanical force into electrical signals and vice versa. When activated by physiological mechanical energy or ultrasound, piezoelectric nanofibers produced through electrospinning can have positive biological effects, including morphological regulation, cell proliferation, and stem cell differentiation [30,41]. Hence, the utilization of electrospun piezoelectric nanofibers for indirect electrical stimulation at cellular and tissue levels has received increasing interest. This study showed that 3 wt.% Cu-nHA composite PVDF-aligned fibers can significantly enhance direct piezoelectric activity through dynamic bending deformation and exhibit antibacterial properties compared with fibers without Cu-nHA. However, this work is limited by the complexity of the instrumentation required to activate these electroactive nanofibers. This complexity hinders a comprehensive understanding of the molecular mechanisms underlying cellular responses to piezoelectric stimulation and the regulation of various cell lineages.

## 4. Conclusions

All the electrospun membranes exhibit an aligned fiber structure. The average fiber diameter in the PVDF/3 wt.% Cu-nHA group is comparable with that of collagen fibers, providing a bionic ligament structure. The F(β) index and piezoelectricity measurements confirm that all the groups possess a piezoelectric β-phase. The PVDF/3 wt.% Cu-nHA group demonstrates the highest piezoelectric value, showing an enhancement of approximately 30% compared with that of the pure PVDF group. This excellent piezoelectricity can enhance ligament tissue regeneration and improve osseointegration at the interface between artificial ligaments and bones. Antibacterial and cytotoxicity tests reveal that the addition of 3 wt.% Cu-nHA significantly boosts the antibacterial effectiveness of the membrane, achieving nearly a 60% reduction in *S. aureus* without inducing cytotoxic reactions. Tensile tests indicate that incorporating Cu-nHA markedly enhances the membrane’s mechanical properties, increasing its tensile stress and maximum strain by about twofold. The PVDF/Cu-nHA membranes meet the deformation needs of the ACL, highlighting its promising potential for both repair and replacement solutions. This study successfully prepared a Cu-nHA/PVDF composite piezoelectric membrane using electrospinning. The addition of 3 wt.% Cu-nHA optimizes the piezoelectricity and mechanical properties of the composite membrane while imparting antibacterial characteristics. The membrane does not exhibit cytotoxicity and shows significant potential for use as an artificial scaffold, with promising applications in ligament tissue engineering.

## Figures and Tables

**Table 1:** Average fiber diameter of electrospun membranes in each group (*n* = 100, *p* < 0.05).

Designated Group	Average Fiber Diameter (nm)
PVDF	645 ± 282
PVDF/1 wt.% Cu-nHA	605 ± 202
PVDF/3 wt.% Cu-nHA	554 ± 126

**Table 2:** Maximum specific voltage recorded from the direct piezoelectric activity of composite membranes under mechanical deformation (*n* = 3; *p* < 0.05).

Groups	Piezoelectric Value (V/g·m^−2^)
PVDF	18.98 ± 1.18
PVDF/1 wt.% Cu-nHA	17.46 ± 0.55
PVDF/3 wt.% Cu-nHA	25.02 ± 0.68

**Table 3:** Mechanical properties to record ultimate tensile stress before breaking and tensile strain after fracture of the electrospun membranes (*n* = 10).

Groups	Ultimate Tensile Stress (MPa)	Strain After Rupture (%)
PVDF	7.60 ± 1.67	16.06 ± 2.92
PVDF/1 wt.% Cu-nHA	13.28 ± 2.84	29.71 ± 12.24
PVDF/3 wt.% Cu-nHA	14.16 ± 2.75	31.33 ± 7.26

## Data Availability

The data presented in this study are available on request from the corresponding author.
